# Draft Genome Sequence of a *Staphylococcus aureus* Strain Isolated from a Cow with Clinical Mastitis 

**DOI:** 10.1128/genomeA.00914-15

**Published:** 2015-08-20

**Authors:** Paresh Sharma, D. Peddi Reddy, P. Anand Kumar, Ramya Gadicherla, Neena George, Vasundhra Bhandari

**Affiliations:** a National Institute of Animal Biotechnology-DBT, Hyderabad, India; b College of Veterinary Science, Sri Venkateswara Veterinary University, Proddatur, Andhra Pradesh, India; c K L University, Vijayawada, Andhra Pradesh, India

## Abstract

We report here the draft genome of *Staphylococcus aureus* causing clinical mastitis in a cow from India. It is a major causative agent of mastitis and, further, livestock-associated strains are emerging as a potential threat to public health, thereby warranting studies to understand the genome of this deadly pathogen.

## GENOME ANNOUNCEMENT

*Staphylococcus aureus* is a highly opportunistic pathogen causing infections in humans and animals. It is majorly responsible for bovine mastitis in animals, which results in a decrease in milk quality and leads to economic losses (1). Further, the zoonotic potential of *S. aureus* strains and the few studies on livestock-associated strains make it crucial to understand the genome of the strain for in-depth analysis (2). The *S. aureus* strain used in this study was isolated from the milk from a cow suffering from clinical mastitis in a dairy herd of India. The genomic DNA was extracted using Wizard genomic kit (Promega) and with the initial addition of lysostaphin (100 µg/ml) and lysozyme (100 µg/ml) for proper lysis of the cells. Multilocus sequence typing (MLST) revealed that the strain belongs to a new group, sequence type 3176 (ST3176) (sequence submitted to http://saureus.mlst.net). Here, we report the draft genome of *S. aureus* strain from a cow infected with mastitis, belonging to a new ST group, 3176.

Genomic DNA was mechanically sheared, and for 300- to 350-bp fragments, paired-end sequencing was done using Illumina MiSeq. Trimming for removing Illumina adapter sequences and removal of low-quality bases were done using the Cutadapt (3) and Sickle (4) softwares, respectively. A total of 4,478,850 reads passed through the quality filter, with an overall G+C content of 37.15%. Draft genome assembly was performed using SPAdes (5) assembler, which predicted the size of the genome to be 2.842 Mb, composed of 87 contigs. Coding sequences (CDSs) were predicted from the contigs using Glimmer (6), which predicted 2,835 coding sequences. The predicted CDSs were compared with NCBI nonredundant protein database using the BLASTx program, which showed similarity to genes present in other fully sequenced *S. aureus* genomes. The predicted CDSs were annotated using the Contig Annotator Pipeline (CANoPI), which considers the best hit (*E* value, ≤10e − 5; similarity score, ≥40%) for functional assignment. BLAST analysis showed closest similarity of the genome with *S. aureus* strain C0673.

This is a first-draft genome sequencing project from India utilizing an *S. aureus* strain isolated from a bovine mastitis case, and the genome analysis will further help us understand the genome of the pathogenic strain.

### Nucleotide sequence accession numbers.

This whole-genome shotgun project has been deposited at DDBJ/EMBL/GenBank under the accession no. LFXB00000000. The version described in this paper is version LFXB01000000.
